# InteliRank: A Four-Pronged Agent for the Intelligent Ranking of Cloud Services Based on End-Users’ Feedback

**DOI:** 10.3390/s22124627

**Published:** 2022-06-19

**Authors:** Muhammad Munir Ud Din, Nasser Alshammari, Saad Awadh Alanazi, Fahad Ahmad, Shahid Naseem, Muhammad Saleem Khan, Hafiz Syed Imran Haider

**Affiliations:** 1 School of Computer Sciences, National College of Business Administration & Economics, Lahore 54700, Pakistan; 82141354@ncbae.edu.pk (M.M.U.D.); rector@ncbae.edu.pk (M.S.K.); 2 Department of Computer Science, College of Computer and Information Sciences, Jouf University, Sakaka 72341, Aljouf, Saudi Arabia; nashamri@ju.edu.sa (N.A.); sanazi@ju.edu.sa (S.A.A.); 3 Department of Basic Sciences, Jouf University, Sakaka 72341, Aljouf, Saudi Arabia; 4 Department of Information Sciences, Division of Sciences and Technology, University of Education, Lahore 54770, Pakistan; shahid.naseem@ue.edu.pk; 5 Department of Software Engineering, University of Lahore, Lahore 54770, Pakistan; imran.haider@se.uol.edu.pk

**Keywords:** cloud services, PaaS, IaaS, SaaS, ranking, machine learning, prediction, classification, sequential minimal optimization regression (SMOreg), multilayer perceptron (MLP), linear regression (LR)

## Abstract

Cloud Computing (CC) provides a combination of technologies that allows the user to use the most resources in the least amount of time and with the least amount of money. CC semantics play a critical role in ranking heterogeneous data by using the properties of different cloud services and then achieving the optimal cloud service. Regardless of the efforts made to enable simple access to this CC innovation, in the presence of various organizations delivering comparative services at varying cost and execution levels, it is far more difficult to identify the ideal cloud service based on the user’s requirements. In this research, we propose a Cloud-Services-Ranking Agent (CSRA) for analyzing cloud services using end-users’ feedback, including Platform as a Service (PaaS), Infrastructure as a Service (IaaS), and Software as a Service (SaaS), based on ontology mapping and selecting the optimal service. The proposed CSRA possesses Machine-Learning (ML) techniques for ranking cloud services using parameters such as availability, security, reliability, and cost. Here, the Quality of Web Service (QWS) dataset is used, which has seven major cloud services categories, ranked from 0–6, to extract the required persuasive features through Sequential Minimal Optimization Regression (SMOreg). The classification outcomes through SMOreg are capable and demonstrate a general accuracy of around 98.71% in identifying optimum cloud services through the identified parameters. The main advantage of SMOreg is that the amount of memory required for SMO is linear. The findings show that our improved model in terms of precision outperforms prevailing techniques such as Multilayer Perceptron (MLP) and Linear Regression (LR).

## 1. Introduction

Cloud Computing (CC) is a form of internet-based computing in which shared, configurable resources are made accessible to computers and other devices as a service. With the increasing popularity of CC, the development of high-quality cloud applications has become a crucial area of research. CC is an architectural style that provides cloud users with on-demand or pay-per-use access to a shared pool of services and computational resources [1,2]. Consumers and businesses can save money on capital expenditures and operating expenses owing to CC. It offers users a network-based environment that enables the sharing of computations and services independent of location [3].

With the increasing popularity of service-computing technologies, cloud services have emerged as essential resources that are commonly employed in the context of software systems. Using a pay-as-you-go concept, customers can select services that are stored in cloud data centers [4]. When selecting a service, users usually prioritize their own unique functional needs. With the increasing scope of services offered by cloud data centers, there may be multiple potential services that meet the customer’s requirements. Due to the fact that these candidate services may all match the functional requirements, the user may be required to evaluate them from a non-functional perspective (considering characteristics such as availability, security, reliability, and cost, etc.) before making a choice. Among all the non-functional indicators of cloud services, reliability is arguably the most important and should thus be the primary factor when selecting services [5,6,7].

CC is a business model that enables convenient, on-demand network access to a shared pool of configurable computing resources (e.g., networks, servers, storage, applications, and services) that can be rapidly provisioned and released with little management effort or interaction from service providers [8]. Through virtualization, CC can support a huge customer base with varying computation needs on the same physical infrastructure. Contrary to prior paradigms, such as clusters and grid computing, CC is service-oriented; it offers virtualized resources on demand as quantifiable and billable utilities [9,10]. Consider one’s experience with email as an example of CC. The email client, whether it be Yahoo!, Gmail, or Hotmail, manages the hardware and software required to operate one’s personal email account. Emails are not stored on someone’s physical computer; they are accessed via an internet connection, which means they are accessible from any location [11,12].

**Figure 1:**
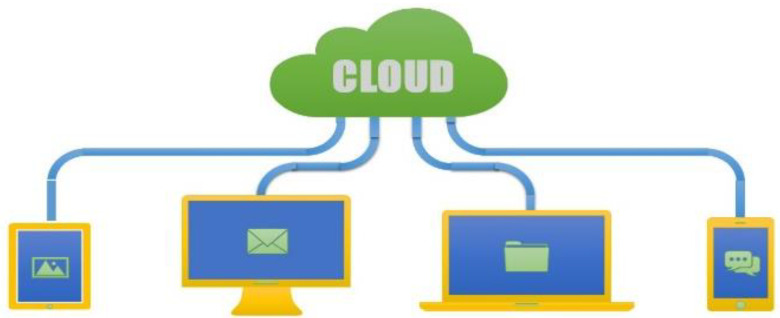
Cloud based computing and communication.

**Figure 2:**
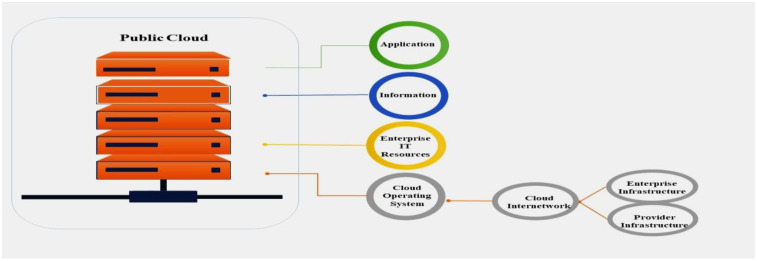
Public cloud computing.

Figure 1 illustrates how, in a CC environment, various accessories such as mobile phones, personal computers/laptops, and servers are used to share information via frame relays, routers, and switches [13,14]. Any subscriber with an internet connection can access the public cloud, as shown in Figure 2. In the simplest terms, public cloud services are those that are made available to clients via the internet by a third-party service provider. The moniker ‘public’ does not always imply that it is free, even if it is possible to utilize it for free or at a low cost. A public cloud does not imply that a user’s data is publicly accessible; public cloud companies often provide means for their users to manage access to their own data [15].

**Figure 3:**
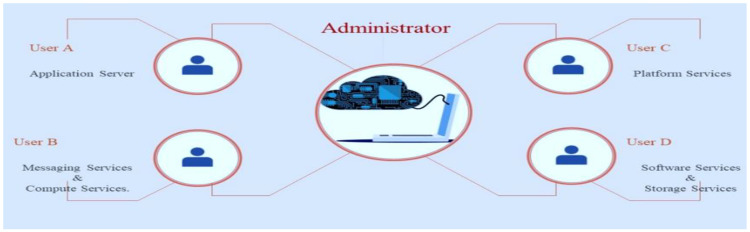
Private cloud computing.

Figure 3 depicts a private cloud that is created for a specific group or organization and restricts access to that group or organization. A private CC environment shares many characteristics with a public CC environment, including adaptability and a service-oriented architecture. The difference between a private cloud and a public cloud is that, with a private-cloud-based service, data and processes are maintained within the organization, without the network capacity, security risks, and regulatory requirements that are associated with public cloud services [16].

**Figure 4:**
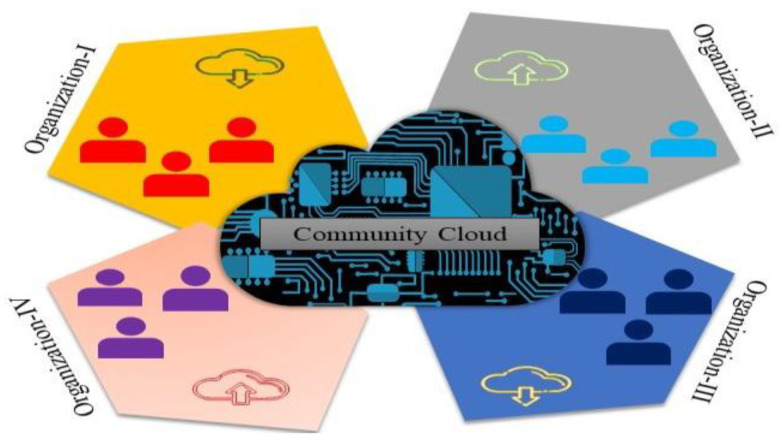
Community cloud computing.

As depicted in Figure 4, a community cloud is a cloud that is shared by two or more enterprises with comparable cloud requirements. A set of organizations with common interests, such as specialized security requirements or a shared mission, will manage and use a community cloud [17].

PaaS, IaaS, and SaaS are the three service types that comprise the CC concept. PaaS is a model in which a third-party provider provides hardware and software tools to customers via the internet. These tools are typically necessary during the application development process. PaaS offers an application’s execution environment, such as the Google App Engine, with point-and-click capabilities that allow non-developers to create their own web applications [18,19]. Second, in IaaS, online services that provide high-level Application Programing Interfaces (APIs) are used to access numerous low-level aspects of the underlying network infrastructure, such as physical computer resources, location, data partitioning, scaling, security, and backup. Thirdly, SaaS enables consumers to access cloud service providers’ applications via a web browser that runs on cloud infrastructure. As a result, end users are not required to download, install, configure, and operate software applications on their personal computing terminals [20,21,22].

Providing rankings involves assigning a value to each option and then arranging them according to that value, with the lowest value representing the best choice. The rank increases as the value decreases. Rankings of cloud services have been gaining popularity over time. However, ranking is subtly different, due to the naming convention and existing cloud infrastructure [23,24]. When there are numerous service providers, selecting a single CC service can be challenging. It is essential to select the optimal ranking methodology and to evaluate all qualitative aspects of the services [25].

In Artificial Intelligence (AI), the creation of a precise prediction model requires the collection of input characteristics that influence cloud-service rankings. However, a significant portion of the required data is inaccessible or incomplete, due to data source retrieval or significant data loss. Therefore, a model with a high degree of accuracy must be created using a limited number of input parameters [26,27]. Various methodologies, including deterministic and probabilistic approaches, have been utilized to estimate the global ranking of cloud services. Few studies have employed LR for estimating cloud service rankings, with multiple LR being the most common technique. This model lacks the ability to comprehend the nonlinearity and complexity of the system’s configuration. To determine the ranking of cloud services, it is preferable to use cutting-edge ML algorithms, which are far more effective and require much less computing time and fewer resources [28,29].

The Machine Learning (ML) tool Waikato Environment for Knowledge Analysis (WEKA) includes an abundance of learning and mining tools. WEKA also makes it easy to test and modify individual learning algorithms to determine which combination of parameters produces the best results [30]. Sequential Minimal Optimization (SMO) is a novel training method for Support Vector Machines (SVM) that divides large problems into as many minor Quadratic Programming (QP) optimization problems as possible [31]. These minor QP issues are resolved analytically, eliminating the requirement for a time-consuming numerical QP optimization in the inner loop. The memory requirements of SMO are proportional to the size of the training set, allowing it to process extremely large training sets, because matrix computation is avoided. SMO scales between linear and quadratic in the training set size for various test problems, whereas the typical SVM technique scales between linear and cubic. SMO can be over a thousand times faster than the typical SVM technique on sparse datasets [32]. For preprocessing, another study utilized wavelet analysis (specifically, the Haar wavelet). It then employed a Support Vector Machine (SVM) via Sequential Minimum Optimization (SMOreg) with Poly kernel function on the updated Quality of Web Service (QWS) dataset to predict cloud-service rankings and model building [33].

The study is organized as follows: Section 1 presents a brief overview of CC, types and uses of CC architectures, developments in the field of CC, and ML techniques for cloud-service ranking using the WEKA tool; Section 2 presents a literature review; Section 3 presents material and methods, Section 4 defines the mathematical modelling, Section 5 includes the experimental results and discussion; Section 6 contains the comparative analysis; and Section 7 concludes the study and results.

### 1.1. Problem Statement

If a user chooses a cloud service from a resource pool of functionally identical services to establish an infrastructure, the service’s credibility is an essential attribute to evaluate, and its rank in the pool reflects the same. However, determining the credibility ranking of a service is not always straightforward. Although user feedback can be used to evaluate the credibility of a service and develop a ranking system, it is heavily influenced by subjective criteria and is quite often biased. Monitoring a service’s QoS, which is represented by its Quality of Service (QoS) properties, is another method for predicting its reliability. Existing methods fall short in terms of generating a good ranking environment for cloud services based on stated factors, necessitating the development of novel validation mechanisms to maintain the system’s transparency and efficiency.

### 1.2. Contribution

A new ranking prediction framework for cloud services is proposed, which integrates a subset of solutions for QP optimization problems. An algorithm for ranking predictions based on the SMOreg method is provided with extensive experiments (on a widely used public QWS dataset of services), which were conducted to validate the framework and techniques presented.

## 2. Literature Review

In this section, a literature review is conducted to shed light on the efforts of various researchers to improve the understanding of CC and related concepts. Several pieces of research on CC and ML algorithms highlight their applications in a variety of fields; these have proven to be an invaluable source of guidance for the presented approach of cloud-service ranking.

According to the study, CC is an internet-centric software model that represents a shift from traditional single-tenant software development to a flexible, multi-tenant, multi-platform, multi-network, global program. This could be as straightforward as a web-based email service or as complex as a globally-distributed load-balanced content-delivery platform. Moreover, the PaaS, SaaS, and IaaS techniques all aim to solve the same scaling issues [34]. Data centers are typically comprised of a large number of interconnected servers that are clustered in densely populated areas where the risk of a catastrophic event is reduced [35].

According to another study, there are only three types of cloud services: SaaS, PaaS, and IaaS, and enormous scalability is required to fit into any of these categories [36]. According to another study, the cloud concept now encompasses what is possible when using web-scale infrastructure on demand, including: managed services, application services, grid computing, software as a service, platform as a service, and anything as a service [37].

Another study developed a cloud business ontology to aid businesses in locating and selecting suitable cloud services. The development of a paradigm that connects a unified business service and cloud ontology enables enterprises to query for the appropriate cloud service. A unified ontology facilitated the cataloging of desired cloud services and the establishment of a connection between business functions and accessible cloud services. This framework could also be utilized as a service repository [38].

In the presented study, the authors propose a ranking-based collaborative filtering strategy for rating movies. Another research group proposed a ranking-oriented strategy for rating books in digital libraries. That study, unlike previous efforts, provided a comprehensive examination of how to accurately rank cloud services, a novel and urgently required research challenge [39]. In a separate study, an agent- and ontology-based technique for discovering optimal cloud services was proposed. Due to the fact that the proposed method is executed before matching the query with the retrieved services, the online search slowed down the process of discovery [40].

A research group presented a prototype for an intelligent cloud-service-discovery system that combines ontology and the mobile agent. Crawlers with knowledge of the structure, location, schema, and other aspects of the cloud data center were anticipated to have direct access. The test has a high degree of precision but a low degree of recall [41]. In another study, researchers created a cloud crawler engine that crawls through various cloud data centers using cloud ontology. The properties of cloud services are documented, and a dataset is established to contain cloud service descriptions. The evaluation indicated, however, that some cloud service information in the dataset, such as name and URL, was not related to semantic meaning [42].

According to the authors, one of the primary objectives of service selection was to provide a fair analogy between the available services so that users could compare and select the services that best meet their needs based on the functional and technical requirements that the services must fulfill. Examples of functional specifications include completed tasks, pricing policies, and services’ domains [43]. Operating system catalogues, single and multiple operating systems support, and cloud services model are all included in the technical specifications [44].

A study explained that Multiple-Attribute Decision Making (MADM) solutions could be applied to ranking predictions and service selections due to their frequent reliance on numerous QoS factors. Resultantly, numerous management and operation science decision models can be applied to the discovery of trusted services [45]. Another study described that, before presenting a comprehensive best-service decision, the Analytic Hierarchy Process (AHP) relied on a survey form to allow various experts to assign weights to QoS criteria for services. AHP, on the other hand, is still susceptible to subjective factors and difficult to automate [46].

The research described that the Preference Ranking Organization Method for Enrichment of Evaluations (PROMETHEE) technique calculated the weight associated with each attribute using the AHP or the Analytical Network Process (ANP), and then ranked the services based on the weights and preferences of the services related to each attribute. PROMETHEE, on the other hand, demanded a number of pairwise comparisons between services and incorporated subjective weights [47]. Similarly, TOPSIS (Technique for Order Preference by Similarity to an Ideal Solution) has sparked a lot of interest in MADM, since it employed entropy to describe the weight of objective QoS features while also taking consumers’ subjective trust preferences into account. Due to its lower uncertainty, PROMETHEE has been found to be superior to AHP and TOPSIS in experiments [48].

Authors described that numerous decision-making techniques conventionally rank cloud service trust preferences. Even if they could predict the creditability of a service, they only employed static criteria or formulas to calculate the trust rate. However, it is sometimes impossible to adequately characterize the extremely complex nonlinear relationship between a service’s reliability and its strengths using simple mathematical models [49]. In addition, the majority of decision-making models struggle to adapt to these dynamic and shifting conditions. AI has been highlighted as a viable solution for subjective, complex, and dynamic problems [50]. Several AI models, such as the Bayes Network, Classification and Regression Trees (CART), and SVM, have poor predictive capabilities, because the link between QoS attributes and trustworthiness in real-world applications is often complex [51]. The improved SVM-r model has more benchmark features that may enhance prediction outcomes [52].

A research group identified that Artificial Neural Networks (ANNs), can effectively represent complex non-linear relationships and thus have the ability to more accurately predict the reliability of cloud services [51,53]. Over the years, advancements in the ANN model have been achieved to deliver a solid prediction method. Recurrent design has substantially improved the effectiveness of neural predictors by only using influential factors as input data. The self-adaptive neuro-fuzzy weighted extreme-learning machine was investigated in order to improve prediction performance [54,55,56].

## 3. Materials and Methods

In the past, the cloud services measurement index was the subject of extensive research and analysis regarding cloud-service ranking. Numerous studies employing diverse methodologies, such as ontology models and agent technology, have been conducted to determine cloud-service ranking. The International Organization for Standardization (ISO) developed the Cloud-Service Ranking Index (CSRI) characteristics, which include accountability, agility, service assurance, cost, performance, security, privacy, and usability. Existing processing methods include pattern matching, which is used to rank cloud services according to functional and non-functional criteria. All relevant sets are retrieved from the cloud storage site and then ranked by the learning module. This study identifies availability, security, reliability, and cost as the most important aspects of cloud-service evaluation. To ensure the long-term viability of the ranking system, cloud service providers must provide both high-performance cloud services and technologically focused features.

**Figure 5:**
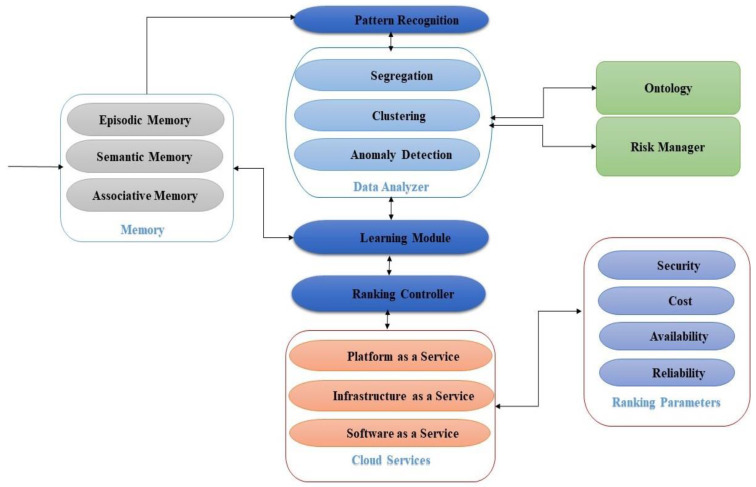
InteliRank: Cloud-service ranking agent.

Figure 5 depicts a CSRA model that identifies the predictors used for comparative cloud-service evaluation. Customers can utilize these metrics to compare various cloud services. The current infrastructure uses the internet to connect various components, but the majority of internet connections are unreliable. Different levels of service quality have been assigned to different customers due to unpredictability, which is one of the primary reasons for the development of a ranking system. CSRA evaluates various cloud services based on user requirements; it is also responsible for quickly and precisely locating and retrieving relevant services based on predefined criteria such as availability, security, reliability, and pricing.

The CSRA receives requests from numerous external users, which may vary based on the requirements of the users, and then examines the requests to identify any abnormalities. If an anomalous request is noticed, it must be sent to the Risk Manager. Otherwise, the agent can search for the user’s requested services and assign a possible service based on the Ranking Parameters if the user’s request is legitimate. The Ranking Parameters must be considered by the Ranking Controller in order to provide the optimal service based on user requirements. The Ranking Controller’s responsibilities include collecting distinctive features for ranking, monitoring the feature’s value, and determining the ranking result.

### 3.1. Memory Module

The memory module is essential, since all other modules and their knowledge patterns are linked to store iteration outputs. All properties and analyses of properties of customers’ homogeneous and heterogeneous QoS requests are retrieved from the memory module, along with the most recent patterns. Memory is a crucial component of cloud-service ranking architecture, since it maintains service classifications, cloud categories, and learning results. This study examined three forms of memory: Episodic Memory (EM), Semantic Memory (SM), and Associative Memory (AM).

#### 3.1.1. Episodic Memory

Every module’s cloud-service-ranking-related actions are saved in EM, which is organized in an episodic fashion. EM describes all of the intended CSRA’s operations and all of the cloud services’ descriptions in episodic stuff, and it is used to record the temporal component of an event.

#### 3.1.2. Semantic Memory

SM is used to record the details of cloud-service patterns, as well as user questions concerning services quality. Availability, flexibility, cost, and security are among the attributes and their functionalities and semantics in QoS. An autonomous system cannot exist until it understands the circumstance, responds appropriately, and moves toward a goal using a strategy. All of these aspects require semantics, and, while the level of expertise of any CSRA is based on time and experience, better and more refined semantics become available as experience accumulated, allowing for a better comprehension of a given circumstance.

#### 3.1.3. Semantic Memory

AM is also required when an agent cannot comprehend the semantics of information and is a crucial component for building relationships between multiple entities, objects, and events. AM examines the specifics of an information pattern and makes associations between various sections of the pattern based on the semantic association of each instance.

### 3.2. Pattern Recognition

Pattern Recognition (PR) is a technique that employs sensory memory to identify data patterns and regularities. Basically, it is the identification of patterns using ML algorithms and sorts of data based on statistical information obtained from patterns and their representation.

### 3.3. Data Analyzer

A Data Analyzer (DA) is a module for analyzing, transforming, and modeling data to unearth pertinent information, make conclusions, and aid in decision making. A DA module can be divided into three categories: segregation, clustering, and anomaly detection.

#### 3.3.1. Segregation

The numerous inputs from online users are collected by our anticipated CSRA. Fixed data-header labels (QoS) and runtime data labels provided by users can both be used to request data input. The CSRA performs a pattern-matching process once the users submit their inputs. Every data label for every piece of user data is checked by the PR unit, which is a separate unit. When every data label has the same data head, the data are homogeneous. On the other hand, the heterogeneous data label provided by the user is compared to known patterns, and their probabilities of match are computed.

#### 3.3.2. Clustering

Clustering is a crucial data-mining and analysis tool for examining online user data trends. Clustering is the process of merging items so that objects within the same group are more similar to one another than objects within other groups. Our CSRA’s objective is to monitor the trend of user inquiries regarding QoS characteristics, such as security, cost, reliability, and availability, and then group these queries based on their similarities.

#### 3.3.3. Anomaly Detection

Theoretically, all security-related attacks are applicable in CC, just as they are in traditional computing. Multiple studies have demonstrated that successful attacks on the infrastructure of Cloud-Service Providers (CSPs) are possible. The proposed CSRA aims to detect heterogeneous data from a large variety of data in a fraction of the time required by current methods. Security entails personal and operational environmental factors that are generally beyond the control of development teams; as a result, a danger must be evaluated and contained through the use of appropriate preventive measures. After successfully detecting heterogeneous data, the CSRA checks for contaminated data and sends it to the Risk Manager (RM) for debugging, if any bugs are found.

### 3.4. Risk Manager

Risks are the potential negative repercussions of using cloud services that could exceed the advantages. The Risk Manager (RM) module uses algorithms to safeguard heterogeneous data, ensuring data privacy and reliability. The Algorithm 1 demonstrates how the risk can be analyzed and managed using diverse security features:
**Algorithm 1.** Risk AnalysisR_i_ = Identified Risksr_f_ = Risk FactorR_netval_ = Monitored Risk Net Value**For** Each Identified Risk R_i_ to be Monitored r_f_**Monitor** the occurrence of related Risk Factor r_1_, r_2_ … r_f_ **For** Each r_f_P (r_f_) = Number of Occurrence/Monitored Time**If** (R_netval_ ≥ R_i_) **Then**Revised the existing Control Strategy with Immediate Action**If** (R_netval_ < R_i_) **Then** no Immediate Action is required

The above task monitors existing risks to ensure they are under control and identifies new risks once the cloud deployment is complete. As user-migrated entities enter the operational phase of risk monitoring, new risk factors may emerge, or the likelihood of existing risk factors may change due to the evolution of cloud platforms, user requirements, or modifications to the CSP’s terms and conditions. As shown in the preceding algorithm, the probability of each R_f_ is determined by its incidence per monitored period. The monitored Risk Net Value (R_netval_) is then determined in the same way as the above-mentioned net-risk computation. If R_netval_ is more than R_f_, we must immediately alter the control strategy; otherwise, we should wait until the next monitoring phase to do so. This is a continuous task that checks in on the status of recognized risks and takes management actions at regular intervals.

### 3.5. Cloud-Service-Ranking Parameter Identification

When a product service is available in the cloud, it means it may be accessed through an interface that links as many users as possible. Cloud security is concerned with network, information, and computer security. To safeguard the data and applications, many strategies and procedures have been implemented. Data protection, identity management, application security, and privacy can all be used to achieve security management. Reliability refers to the system’s capacity to perform the required functionality over a set length of time and the system’s ability to revert to its previous state once a problem occurs. The cloud’s cost approach is more flexible and metered, with users paying only for what they use. Users of cloud services can compare the multiple parameters of CSPs in terms of performance. Algorithm 2 describes the procedure of CSR by utilizing the information regarding main categories, sub-categories, users’ queries, and optimized parameters for ranking the cloud services in an existing category or developing a new category.
**Algorithm 2.** Categorization of Cloud Services**Input:** Customer Cloud Services Preferences with their Queries**Output:** Cloud Services Ranking**Cloud Services Provider Main Categories []** = x_1_, x_2_, x_3_, … x_i_ …, x_n_**Sub-Categories** = c_ij_
∈ x_i_**Cloud Services Ranking System [x_i_]** = Nil**Main Categories (mc)**∈ PaaS, IaaS, SaaS**Sub Category [sc]**∈ Business Application Platform, Raw Computing Platform, Web Hosting, Databases, Open Cloud Platform, Web Application Platform, Application Hosting, Storage, Networking and Infrastructure Service Management.**Cloud Services Mapping [x_i_]** = y_i_**Learning Category [lc]**∈ **New Category [nc]****Begin****Extract** queries (Customer Cloud Preferences)**For** each CSP List [x_i_] do          //CSP = Cloud Services Provider**For** each CSP Category [i] go**If** EQ = CSP Description [i]          //EQ = Extract Query**Bind** Service Sub Category [ij] = EQ**Bind** Cloud Services Ranking [x_i_] ∈ Service Main Category [mc]**Bind** Service Sub-Category [sc] ∈ Service Main Category [mc]**Else**CSM          //Cloud Services Mapping**Function** Cloud Services Mapping [x_i_]**For** each in Cloud Category [x_i_]**Mapping** Cloud Services [y_i_]**End For****If** Service Sub-Category [ij] = match **Then**CSRA [x_i_] **For** each Service Sub-Category [ij]          //Cloud Services Ranking**Else**Learning Category [lc] ∋ [sc]**Function** Learning Category [lc]**End If****End For****If threshold** > **Max** Sim [lc] **Then****Bind** CSRA [x_i_] ∈ Service Main Category [i]**Bind** Main Category [x_i_] ∈ Service Sub-Category [ij]**Else****If Threshold** > **Max** Sim (nc) **Then****Bind** Register New Category [nc] ∈ Service Sub-Category [ij]**Bind** Service Sub-Category [ij] ∈ Service Main Category [x_i_]**Else****Learning** New Cloud Category (nc)**Hold** Learned Cloud Category (lc)**End If**

### 3.6. Learning Module

CSRA is created through iterations, with each iteration involving the extraction and optimization of new attributes. The Learning Module (LM) is a ML-based technique that generates an ontology for patterns using specified parameters. During the initial iteration, supervised learning occurs, and the number of events increases until a certain threshold is reached, at which point semi-supervised learning takes place, followed by unsupervised learning. If such patterns are rich enough to classify a service using similarity analysis, the threshold for adding new cloud services to the LM can be validated. In the LM, all extracted cloud services can be compared, and a predetermined threshold can be used to determine the primary categories of SaaS, PaaS, and IaaS. After identifying the primary category, the LM checks for sub-categories. The process of detecting and validating cloud services is carried out by the LM using the learning algorithm. Each set in the LM contains the cloud semantic concept, its description, service-level-agreement (SLA) information, similarity terms, and source information for the purposes of building and mapping.

An already defined cloud service category or generating a new category exit in both cases; if the learning trend is less than a threshold level throughout the learning process, it can be held for any future learning. The hold category must be trained enough to select an already-defined category or a new category in the cloud evaluation system once it reaches the threshold level. The implementation of the LM is described in Algorithm 3, i.e., how it learns the similarity among the specific cloud services and assigned them existing or new categories as well as sub-categories:
**Algorithm 3.** Learning Management for Cloud Services Categorization Learning for Main Category
**Check** Similarity ← Sim (Provider_1_, Provider_2_, Provider_n_)**Max** Similarity α (SM_1_, SM_2_, SM_n_)  ϵ Main Category Cloud RelevanceSM (IaaS, SaaS, PaaS) ∈ **Max** Similarity**Update** Cloud Repository ←|Category|+|Max Similarity|
 Learning for Sub Category
5.Check Similarity ← Sub-Category (Storage, Compute, Network … n)6.Max Similarity α (SM1, SM2, SMn) ∈ Main Category Cloud Relevance7.Sub-Category ∈ Max Similarity Sub-Category8.Update Cloud Repository ← | Sub-Category|+| Max Similarity|


In summary, the anticipated CSRA model receives inputs regarding cloud services via users’ feedback, identifies some dynamically optimized parameters, and predicts the relative ranking of the available cloud services. The CSRA can assist in providing real-time and trustworthy options to users based on their needs.

## 4. Mathematical Modelling for Sequential Minimal Optimization

Using ML approaches for ranking prediction aims to create a model automatically, based on the training dataset. SVM was utilized to implement the learning method. Using data-mining techniques, datasets can be effortlessly preprocessed, classified, and predicted. In this research, the learning algorithm employs a unique approach of SVM using SMOreg and Poly kernel (PolyKernel). With considerable success, the SMOreg learning algorithm is used to accumulate knowledge in expert systems. This method reduces the RMSE so that the projected ranking of cloud services is identical to the observed ranking.

Sequential Minimal Optimization (SMO) is a simple approach for quickly solving the SVM Quadratic Programming (QP) problem without the need for any additional matrix storage or numerical QP optimization steps. SMO decomposes the overall QP problem into QP sub-problems, ensuring convergence with Osuna’s theorem. Unlike earlier methods, SMO chooses to address the smallest optimization problem possible at each phase. Due to the fact that the Lagrange multipliers must follow a linear equality requirement, the shortest possible optimization issue for the basic SVM QP problem contains two Lagrange multipliers. SMO selects two Lagrange multipliers to jointly optimize at each step, discovers their optimal values, and updates the SVM to reflect the new optimal values. The advantage of SMO is that it does not require any additional matrix storage. As a result, even very large SVM training problems can fit into the RAM of a standard computer or workstation. SMO is less prone to numerical precision issues, because it does not employ matrix methods. SMO consists of two parts: an analytic method for determining the two Lagrange multipliers and a heuristic for determining which multipliers to optimize.

SMO solves for the two Lagrange Multipliers (LMP) by first computing the constraints on them and then solving for the constrained minimum. All quantities referring to the first multiplier have a subscript a, and all quantities referring to the second multiplier also have a subscript b for ease. The limitations may simply be shown in two dimensions, because there are only two LMPs.

The extremities of the diagonal-line segment are easily generated. The algorithm computes the second LMP α*_b_* and the extremities of the diagonal line segment in terms of α_b_ without losing generality. The following constraints apply to α*_b_* if the target *t_a_* does not equal the target *t_b_*:

(1) $$LMP\left(low\right)=max\left(0,\alpha_{b}-\alpha_{a}\right)\;\;\;\;\;\;\;\;\;\;\;\;\;\;\;\;\;\;\;LMP\left(high\right)=min\left(D,D+\alpha_{b}-\alpha_{a}\right)$$

If the target *t_a_* equals the target *t_b_*, then the following constraints apply to α*_b_*:

(2) $$LMP\left(low\right)=max\left(0,\alpha_{b}+\alpha_{a}-D\right)\;\;\;\;\;\;\;\;\;\;\;\;\;\;\;\;\;\;\;\;\;LMP\left(high\right)=min\left(D,\alpha_{b}+\alpha_{a}\right)\;\;$$

The objective function’s second derivative along the diagonal line can be represented as:

(3) $$\mu =Ker\left(\vec{y_{a}},\vec{y_{a}}\right)+Ker\left(\vec{y_{b}},\vec{y_{b}}\right)-2Ker\left(\vec{y_{a}},\vec{y_{b}}\right),\;\;\;$$

where he goal function must be positive and have a minimum along the direction of the linear equality constraint, and μ must be greater than zero in normal situations. In this scenario, SMO calculates the minimum along the constraint’s direction:

(4) $$\alpha_{b}^{new}=\alpha_{b}+\frac{t_{b}\left(Er_{a}-Er_{b}\right)}{\mu },$$

where $Er_{i}$ is the error on the ith iteration. Then constrained minimum is found by extracting the unconstrained minimum to the ends of the line segment:

(5) $$\alpha_{b}^{new,extracted}=\{\begin{array}{lll} LMP(high) & if & \alpha_{b}^{new}\geq LMP(high)\\ \alpha_{b}^{new} & if & LMP(low)<\alpha_{b}^{new}<LMP(high)\\ LMP(low) & if & \alpha_{b}^{new}\leq LMP(low) \end{array}$$

Now, let *v* = *t*_1_*t*_2_ then the value of $\alpha_{a}$ is computed from the $\alpha_{b}^{new,extracted}$:

(6) $$\alpha_{a}^{new}=\alpha_{a}+v\left(\alpha_{b}-\alpha_{b}^{new,extracted}\right)\;\;\;\;\;$$

μ might not be positive in unusual cases. If the kernel Ker does not obey Mercer’s condition, a negative μ might occur, causing the objective function to become indefinite. If more than one training sample has the same input vector x, a zero μ can occur even with a valid kernel. In any instance, SMO can operate even if μ is negative, in which case the objective function σ should be evaluated at each line segment’s end:

(7) $$g_{1}=t_{a}\left(Er_{a}+\varepsilon \right)-\alpha_{a}Ker\left(\vec{y_{a}},\vec{y_{a}}\right)-v\alpha_{b}Ker\left(\vec{y_{a}},\vec{y_{b}}\right)\;\;\;\;\;\;$$

(8) $$g_{2}=t_{b}\left(Er_{b}+\varepsilon \right)-v\alpha_{a}Ker\left(\vec{y_{a}},\vec{y_{b}}\right)-\alpha_{b}Ker\left(\vec{y_{b}},\vec{y_{b}}\right)\;\;\;\;\;$$

(9) $$LMP{\left(low\right)}_{a}=\alpha_{a}+v\left(\alpha_{b}-LMP\left(low\right)\right)\;\;\;\;$$

(10) $$LMP{\left(high\right)}_{a}=\alpha_{a}+v\left(\alpha_{b}-LMP\left(high\right)\right)\;\;\;\;\;\;\;\;\;\;$$

(11) σLMP(low)=LMP(low)a.g1+LMP(low)g2+12LMP(low)a2 Ker(ya→,ya→)+12LMP(low)2 Ker(yb→,yb→)+vLMP(low)LMP(low)aKer(ya→,yb→)  

(12) σLMP(high)=LMP(high)a.g1+LMP(high)g2+12LMP(high)a2 Ker(ya→,ya→)+12LMP(high)2 Ker(yb→,yb→)+vLMP(high)LMP(high)aKer(ya→,yb→) 

The Lagrange multipliers can be moved by SMO to the end point with the lowest objective function value. The joint minimization cannot progress if the objective function is the same at both ends with minimum ε error and the kernel obeys Mercer’s requirements.

## 5. Experimental Results and Discussion

Experiments were carried out on a Lenovo Mobile Workstation equipped with the Processor: 11th Generation Intel Core i9, Operating System: Windows 10 Pro 64, Memory: 128 GB DDR4, Hard Drive: 1 TB SSD, Graphics: NVIDIA RTX A4000. We used the Prism-GraphPad 9.4.0 and WEKA 3.9.6 tools for the explanation and results of our proposed scheme, and the language used in it is Java. Two traditional methods (MLP and LR) were also evaluated and compared to the SMOreg method.

This study included 2283 instances that contain information about different CSPs found in the QWS dataset. The services are ranked into seven different categories based on their performance obtained through the selected parameters, as listed in Table 1.

**Figure 6:**
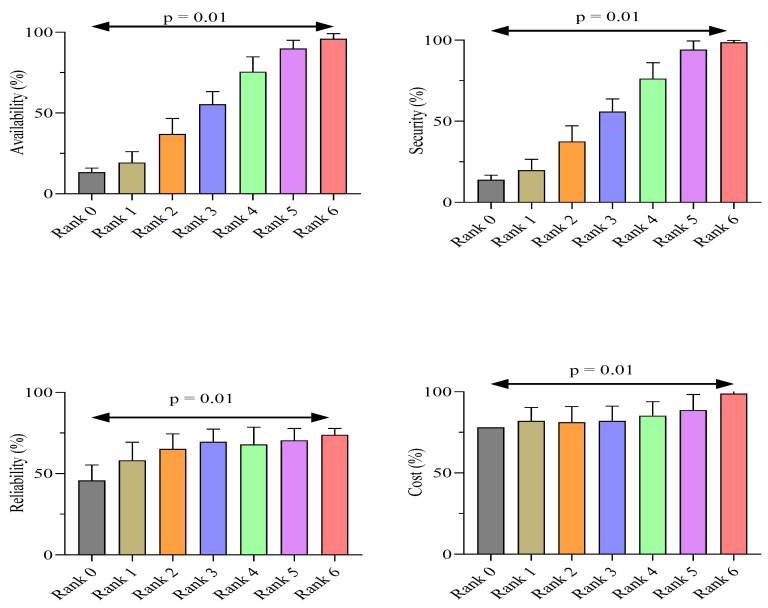
Cloud-service ranking for identified parameters using Student’s *t*-test.

The results of independent Student’s *t*-test indicate significant mean differences for availability, reliability, security, and cost according to the ranking of cloud services as shown in Figure 6. Rank 0 has lowest mean for all these variables, and rank 6 shows the highest means, as presented in the above graph. The anticipated CSRA behaves differently and dynamically due to the identified significant difference, which cannot be accepted as a normal variation.

**Figure 7:**
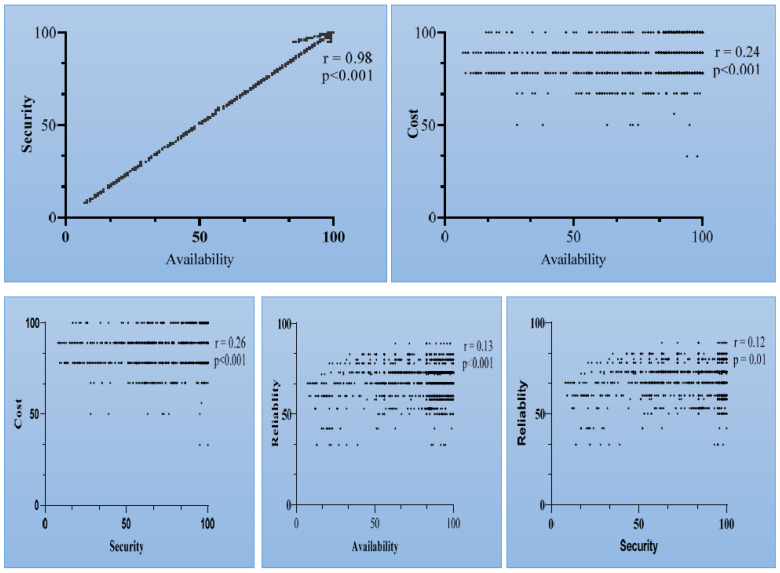
Correlation analyses among identified parameters using end-users’ feedback.

Correlation analysis was performed to evaluate if there any significant relationships among the identified variables obtained through the end-users’ feedback, as shown in Table 2. The results of our analysis indicate a strong relationship between security and availability (r = 0.98, *p* < 0.01). However, a significant but weak relationship was observed between cost and availability (r = 0.24, *p* < 0.01). Similar findings were observed for cost with security, reliability and security. A positive correlation coefficient denotes that the value of one variable is directly dependent on the value of the other variable. The results are indicated in the Figure 7.

### 5.1. Cloud-Service Ranking

#### 5.1.1. Sequential Minimal Optimization Regression (SMOreg)

The SMOreg scheme is employed to rank cloud services based on the identified attributes. SVM for regression is implemented in SMOreg. The parameters are learnable through a variety of methods. The focus of the SMOreg is cloud-service ranking for competitiveness prediction. The RegOptimizer controls which algorithm is used. SMOreg used a dataset: {(x_1_, y_1_), …, (x_n_, y_n_)}, where x_i_ are input vectors and y_i_ are the scalar target outputs denoting ranking classes (R_1_, R_2_, …, R_7_). The two-layer architecture contains an input layer of four inputs. Then, there are a total of seven SVMs in SMOreg dented by S_g_, each one learning to extract one latent variable f(x|*l*)_g_ from an input pattern x. Here *l* denotes the trainable parameters in the hidden-layer SVMs. Finally, there is the main SVM in SMOreg denoted by M_g_ that learns to approximate the target function using the extracted feature vector as an input. The total number of correct instances divided by the total number of instances provides the accuracy of the anticipated model SMOreg. This technique is only applicable if the amount of labeled data is insufficient or if application or device-specific solutions are required to improve the accuracy of detection. Table 3 shows an optimized SMOreg characterization for cloud-service classification. The accuracy rate obtained is 98.71 per cent. The prediction speed was roughly 110 observations per second, and the training time was 3.138 s. We made a comparison of two of the most widely used and well-known algorithms versus multiclass classification (i.e., the MLP and LR).

The disparity between the estimates and the actual results is measured using the Mean Absolute Error (MAE):

(13) $$\mathrm{MAE}=\frac{1}{n}\overset{n}{\underset{i=1}{\sum }}\left|o_{i}-t_{i}\right|$$

The MAE is an average of the absolute errors, where $o_{i}$ is the estimate and $t_{i}$ the true value as shown in (13).

The sample standard deviation of the variations between anticipated and true values is represented by the Root Mean Squared Error (RMSE) in (14):

(14) $$\mathrm{RMSE}=\sqrt{\frac{1}{n}\overset{n}{\underset{i=1}{\sum }}{\left(o_{i}-t_{i}\right)}^{2}}\;\;\;\;\;$$

We utilized C = 1 to evaluate the RegSMOImprove model type and tweak its respective parameters, achieving a minimum MAE and RMSE. Due to the fact that the minimum MAE and RMSE are determined for the exponent of 1, the performance of the RegSMOImprove model and adjustment of the exponent parameter revealed that cloud-service data are simple in nature. This means that lines can be used to segregate data. The MAE and RMSE did not change much when the parameter of the PolyKernel function was changed. We came to the conclusion that the performance parameters of the various kernel models differ slightly. PolyKernel outperforms the other two by a small margin. As a result, the PolyKernel function was employed for the SMOreg classifier.

#### 5.1.2. Multilayer Perceptron (MLP)

The MLP is used to make detailed predictions of the input parameters. Table 4 shows the MLP characterization, which shows that a precision rate of 98.02 % was achieved. The MLP is used with a 2.5 scaling rate and a one-to-many criterion.

#### 5.1.3. Linear Regression (LR)

Here, the LR with M5 technique is used, which may yield a more precise rate (based on the data). The LR details are listed in Table 5, which includes a precision rate of 71.4%.

**Figure 8:**
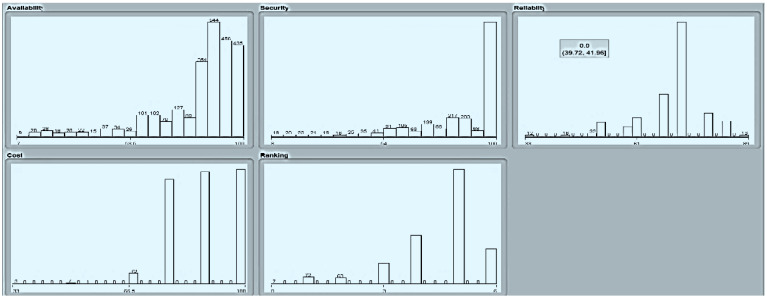
Parametric distribution of dataset.

**Figure 9:**
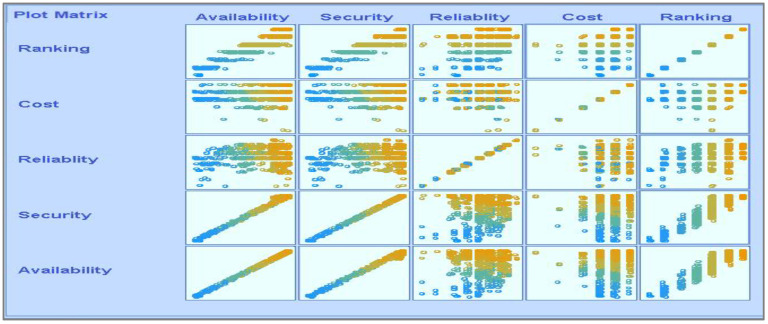
Combined matrix plot.

**Figure 10:**
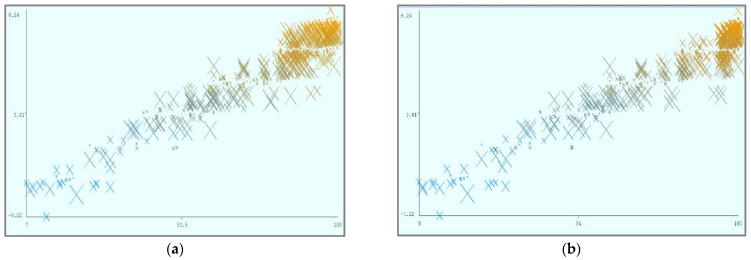

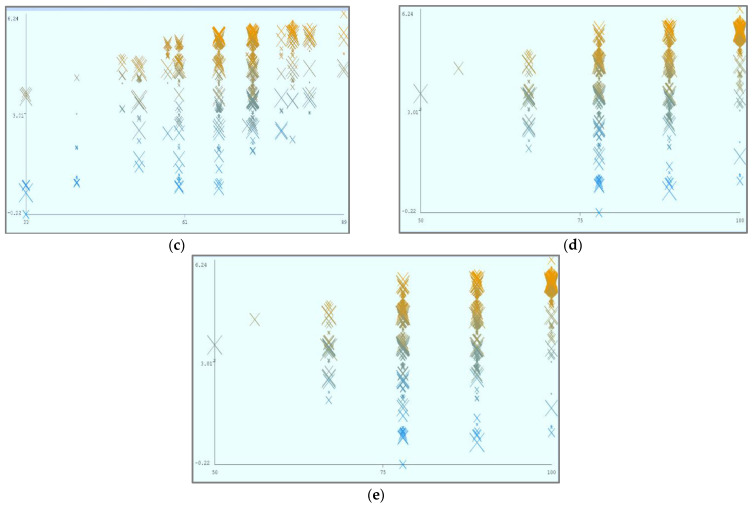
(**a**). Sequential minimal optimization regression error (X: Availability vs. Y: Predicted Ranking). (**b**). Sequential minimal optimization regression error (X: Security vs. Y: Predicted Ranking). (**c**). Sequential minimal optimization regression error (X: Reliability vs. Y: Predicted Ranking. (**d**). Sequential minimal optimization regression error (X: Cost vs. Y: Predicted Ranking). (**e**). Sequential minimal optimization regression error (X: Ranking vs. Y: Predicted Ranking).

**Figure 11:**
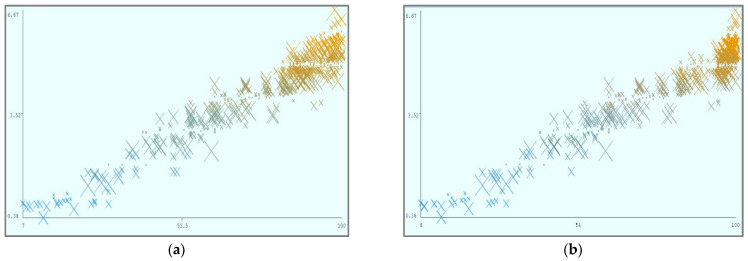

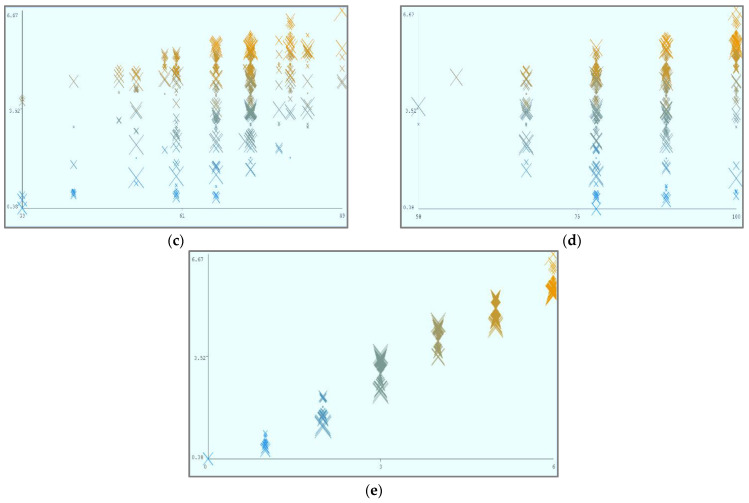
(**a**). Multilayer Perceptron error (X: Availability vs. Y: Predicted Ranking). (**b**). Multilayer Perceptron error (X: Security vs. Y: Predicted Ranking). (**c**). Multilayer Perceptron error (X: Reliability vs. Y: Predicted Ranking). (**d**). Multilayer Perceptron error (X: Cost vs. Y: Predicted Ranking). (**e**). Multilayer Perceptron error (X: Ranking vs. Y: Predicted Ranking).

**Figure 12:**
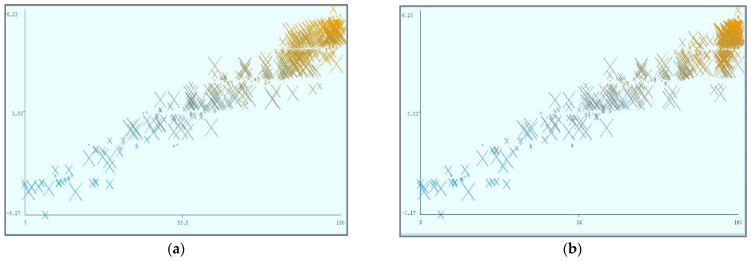

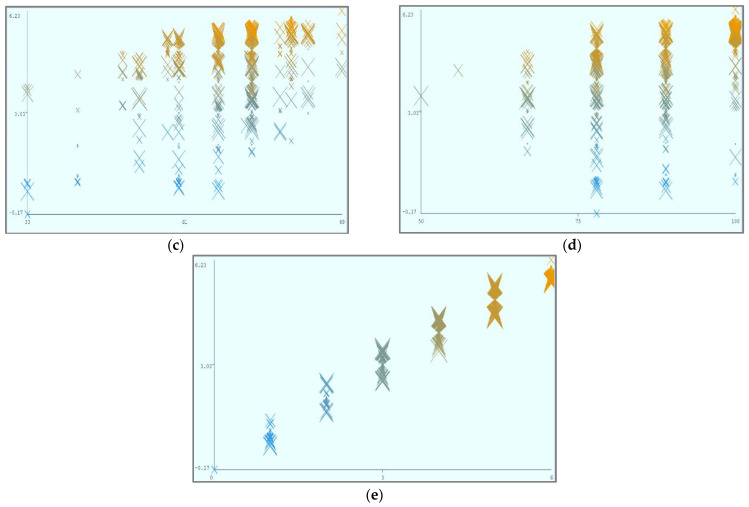
(**a**). Linear regression error (X: Availability vs. Y: Predicted Ranking). (**b**). Linear regression error (X: Security vs. Y: Predicted Ranking). (**c**). Linear regression error (X: Reliability vs. Y: Predicted Ranking). (**d**). Linear regression error (X: Cost vs. Y: Predicted Ranking). (**e**). Linear regression error (X: Ranking vs. Y: Predicted Ranking).

Figure 8 shows the parametric distribution of the dataset with the same scale. In addition, Figure 9 illustrates the combined matrix plot to indicate the relationships among the identified parameters. The error plots from Figure 10a–e were acquired through SMOreg after performing the cloud-service-ranking prediction via the defined input parameters. There were 2283 instances in the dataset, as shown in Figure 9, comprising availability (7–100), security (8–100), reliability (33–89), and cost (33–100) for cloud-service ranking (0–6) data samples. The SMOreg results reveal that the accuracy rate for the 5.0 services ranking is the best, with 1127 samples with 6 misclassified cases. The 6.0 services ranking places it in the second tier (819 correctly classified samples and 4 misclassified cases). The 4.0 services ranking is now in third place (112 correctly classified cases and 3 misclassified cases). The 3.0 services ranking is now in fourth place (105 correctly classified cases and 4 misclassified cases). The 1.0 services ranking is currently ranked fifth (40 correctly classified cases and 3 misclassified cases). The 2.0 services ranking is now at sixth place (35 correctly classified cases and 2 misclassified cases). Finally, with 1 correctly identified, the 0.0 services ranking is in last place. In the 5.0 services ranking, the MLP had the best accuracy rate, with 1096 correctly categorized cases and seven misclassified samples, although the overall misclassification rate was higher than that for SMOreg. Similarly, the 5.0 services ranking obtained the highest accuracy rate in the LR, with 988 correctly categorized cases and eight misclassified cases, but the overall misclassification rate was significantly higher than the previous two MLP and LR. In addition, Figure 11a–e and Figure 12a–e were acquired through MLP and LR, respectively, after performing the cloud-service ranking prediction using the identified input parameters.

Using the abovementioned parameters, different visualizations of cloud-service ranking can be achieved. In (a), Availability (X) and Reliability (Y) remain static, and Ranking (Z) is dynamic. In (b), Availability (X) and Security (Y) remain static, and Ranking (Z) is dynamic. In (c), Reliability (X) and Security (Y) remain static, and Ranking (Z) is dynamic. In (d), Cost (X) and Security (Y) remain static, and Ranking (Z) is dynamic.

To improve the performance of the chosen technique, SMOreg, various hyperparameters such as batch size, C value, filter type, kernel size, and regression optimizer were investigated. On the training set, the model was trained using the cross-validation setup. As a performance metric, the accuracy of each set of hyperparameters in the SMOreg model was employed. The input layer had four nodes, while the output layer had seven nodes, one for each of the seven classes.

When assessing the effect of the SMOreg model on classification performance, five factors were taken into account: batch size, C value, filter type, kernel size, and regression optimizer. The numbers 1 to 21 in the first column represent 21 possibilities, while the second column reflects the selected configuration, as shown in Table 6. The five factors are represented by the third to seventh columns. An instance is represented by a row in the table. The batch size is 100, the C value is 1.0, the filter type is normalization, the kernel is PolyKernel, and the repression optimizer is RegSMO-optimized in the fifth configuration, for example. The observed and predicted rankings in the seventh and eighth columns represent the model’s classification performance using the provided hyperparameters.

It is evident from Table 6 that the classifier SMOreg perform well, as the accuracy measure is ideal and consistent throughout. When the network depth is increased within the defined range, the accuracy improves. However, as the network moves further away from the defined range, the accuracy drops, indicating that too many parameters in the network may cause overfitting and reduce generality. With increasing kernel size, the accuracy measure rises considerably before falling. When the batch size is 100, the highest accuracy measure appears, and the accuracy measure rises as the learning rate rises from 0.00001 to 0.0005. As a result, Table 6 displays the optimal configuration combination. Table 3 lists the optimal settings based on the previous work. These hyperparameters were used to train a new SMOreg model. The classification accuracy of the model is 98.71%, indicating that this choice of hyperparameters is the best. Furthermore, the suggested SMOreg framework takes 3.138 s, which is quick and suitable for real-time applications.

The exploratory findings illustrate that the SMOreg approach is more precise and resilient than existing prediction models, and that it can subvert the dataset’s latent feature characterizations and aptly categorize them. As mentioned above, the ranking of cloud services was accomplished using the identified characteristics and the SMOreg classifier. Remarkably, the accuracy rate was 98.71 percent, which may be due to the exact nature of the values found in QWS dataset.

#### 5.1.4. Cloud-Service Ranking through K-Means Clustering

**Figure 13:**
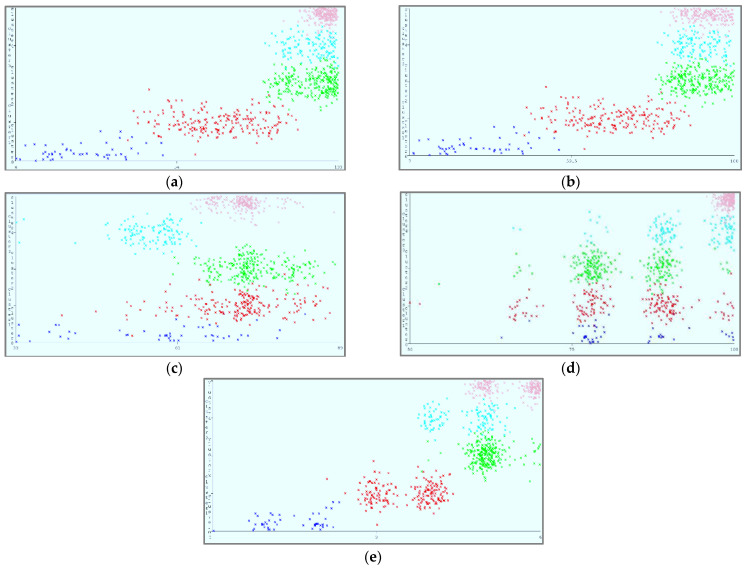
(**a**). Clustering based on availability through self-organizing map. (**b**). Clustering based on security through self-organizing map. (**c**). clustering based on reliability through self-organizing map. (**d**). Clustering based on cost through self-organizing map. (**e**). Clustering based on ranking through self-organizing map.

Figure 13a–e shows the result of the SOM, which represents the classification of cloud services based on the identified criteria while operating in a cloud environment, assuming that one parameter remains constant. The small clusters, represented with different colors, show different categorizations of services with specific clusters. The K-means method was used to partition the clusters, resulting in the formation of five clusters, as shown in Table 7. Cluster membership is shown by different colors. A great degree of the availability, security, and reliability states with low costs indicates high-ranked cloud services. In contrast, a low degree of the availability, security, and reliability states with high costs indicates low-ranked cloud services. However, a moderate degree of the availability, security, and reliability states with moderate costs indicates moderate-ranked cloud services. The maps depict different categorizations of cloud services based on fluctuations in the identified parameters. Clusters’ density characterizations are shown in Table 8.

## 6. Comparative Analysis

Table 9 provides a comparison of the proposed effort with a few previous similar types of research. 

## 7. Conclusions, Limitations, and Future Work

CC has emerged as a key paradigm for enterprises to outsource their different IT needs. CC, also known as PaaS, IaaS, SaaS, and other services, is a technology that provides everything as a service on demand over the internet. There is currently a plethora of CSPs offering various cloud services with varying functionalities. Due to the fact that there is no standard for describing cloud services, each cloud provider describes and expresses cloud services in its own manner. With the increasing number of cloud-based services, each one enables users to access the cloud’s nearly limitless computing resources. It has become increasingly difficult for cloud consumers to identify the best cloud services that meet their QoS requirements. In order to choose between various cloud services, customers must have a method for identifying and measuring the critical performance characteristics of their applications. It is challenging to locate cloud services represented by numerous concepts. As a result, the CSRA established a paradigm based on the functional characteristics of cloud services. The goal of this CSRA is to identify the QoS ranking parameters such as availability, security, reliability, and cost that can be used to compare different cloud services. In this context, the work presented for cloud-service ranking systematically identifies all of the QoS parameters and ranks cloud services based on these identified parameters. We presented a ranking mechanism based on comparisons that may evaluate cloud services via several ranking metrics. By offering a uniform mechanism to evaluate the relative ranking of cloud services, our anticipated CSRA also tackles the difficulty of varying dimensional units of multiple QoS ranking metrics. We also provided a comparison of our proposed CSRA with currently utilized cloud-service ranking systems. The results of the evaluation reveal that our proposed CSRA is both feasible and consistent. We conducted extensive experiments on a well-known, publicly available QoS-based QWS dataset to validate the efficacy of the anticipated strategies. The experimental results demonstrate that the SMOreg technique is more accurate and robust than existing estimation methods and can exploit the dataset’s latent feature descriptions and accurately classify them. Cloud-service ranking was achieved using the identified attributes and the SMOreg classifier. Surprisingly, the accuracy rate was 98.71%, which is higher than the other two approaches, LR and MLP, which had accuracies of 71.4% and 98.02%, respectively. 

Despite the fact that our proposed SMOreg method has a high prediction rate of 98.71 percent, there are still unresolved concerns that necessitate further study. Our current model applies only objective and exact QWS values, despite the fact that QWS measures may be more subjective in certain real-life situations. 

In the future, we can also investigate methods to forecast rankings based on mixed forms of QWS values. Moreover, because the QoS of a service may change over time, it is necessary to dynamically assess the service’s ranking.

## Figures and Tables

**Table 1:** Ranking Distribution with Percentages.

Rank	Number of Instances	Percentage
**0**	7	0.31%
**2**	72	3.15%
**3**	63	2.76%
**4**	526	23.04%
**5**	1239	54.27%
**6**	376	16.47%
**Total**	**2283**	**100.00%**

This table shows that most of the cloud services are ranked into the category of 5.

**Table 2:** Correlation Analysis.

		Cost	Reliability	Security	Availability
**Availability**	PC	0.244	0.13	0.98	1
	*p*-value	<0.001	<0.001	<0.001	
**Security**	PC	0.26	0.12	1	
	*p*-value	<0.001	0.01		
**Reliability**	PC	−0.03	1		
	*p*-value	0.13			
**Cost**	PC	1			
	*p*-value				

**Table 3:** Sequential minimal optimization regression characterization.

Parameter	Value
Correlation Coefficient	0.9656
Mean Absolute Error	1.2524
Root Mean Squared Error	1.2948
Relative Absolute Error	28.9933%
Root Relative Squared Error	26.0872%
Total Number of Instances	852 (60%)
Accuracy Rate	98.71%
Prediction Speed	~110 Obs/S
Training Time	3.138 S
Model Type	Regsmoimprove
Function	Sequential Minimal Optimization Regression

**Table 4:** Multilayer Perceptron Characterization.

Parameter	Value
Correlation coefficient	0.5697
Mean absolute error	1.9282
Root mean squared error	1.9765
Relative absolute error	36.2153%
Root relative squared error	34.4743%
Total Number of Instances	852(60%)
Accuracy	98.02%
Prediction Speed	~111 obs/s
Training Time	0.8658 s
Model Type	Feedforward Neural Network
Function	Multilayer Perceptron

**Table 5:** Linear Regression Characterization.

Parameter	Value
Correlation coefficient	0.9658
Mean absolute error	13.253
Root mean squared error	3.2931
Relative absolute error	29.067%
Root relative squared error	25.9384%
Total Number of Instances	852(60%)
Accuracy	71.4%
Prediction Speed	~881 obs/s
Training Time	0.5868 s
Model Type	M5 Method
Function	Linear Regression

**Table 6:** Comparison of the Configuration Results.

Configuration Number	Instance Number	Batch Size	C	Filter Type	Kernel	Regression Optimizer	Observed Ranking	Predicted Ranking
**1**	88	100	1.0	Normalization	PolyKernel	RegSMOOptimized	0.0	~0.02
**2**	121	100	1.0	Normalization	PolyKernel	RegSMOOptimized	0.0	~−0.09
**3**	71	100	1.0	Normalization	PolyKernel	RegSMOOptimized	0.0	~−0.21
**4**	287	100	1.0	Normalization	PolyKernel	RegSMOOptimized	1.0	~0.67
**5**	212	100	1.0	Normalization	PolyKernel	RegSMOOptimized	1.0	~0.68
**6**	113	100	1.0	Normalization	PolyKernel	RegSMOOptimized	1.0	~0.65
**7**	426	100	1.0	Normalization	PolyKernel	RegSMOOptimized	2.0	~1.85
**8**	315	100	1.0	Normalization	PolyKernel	RegSMOOptimized	2.0	~1.80
**9**	270	100	1.0	Normalization	PolyKernel	RegSMOOptimized	2.0	~1.65
**10**	89	100	1.0	Normalization	PolyKernel	RegSMOOptimized	3.0	~2.77
**----------------------------------------------------**								
**11**	46	100	1.0	Normalization	PolyKernel	RegSMOOptimized	3.0	~2.84
**12**	3	100	1.0	Normalization	PolyKernel	RegSMOOptimized	3.0	~2.74
**13**	251	100	1.0	Normalization	PolyKernel	RegSMOOptimized	4.0	~3.56
**14**	217	100	1.0	Normalization	PolyKernel	RegSMOOptimized	4.0	~3.85
**15**	164	100	1.0	Normalization	PolyKernel	RegSMOOptimized	4.0	~3.95
**16**	10	100	1.0	Normalization	PolyKernel	RegSMOOptimized	5.0	~5.19
**17**	39	100	1.0	Normalization	PolyKernel	RegSMOOptimized	5.0	~5.43
**18**	63	100	1.0	Normalization	PolyKernel	RegSMOOptimized	5.0	~5.19
**19**	9	100	1.0	Normalization	PolyKernel	RegSMOOptimized	6.0	~5.64
**20**	28	100	1.0	Normalization	PolyKernel	RegSMOOptimized	6.0	~5.78
**21**	47	100	1.0	Normalization	PolyKernel	RegSMOOptimized	6.0	~5.76

**Table 7:** Clustering Based on Characterization.

Attributes		Cluster				
		0 (0.05)	1 (0.23)	2 (0.33)	3 (0.14)	4 (0.25)
**Availability**	Mean	24.6357	64.3322	90.0137	89.2023	92.525
	Standard Deviation	9.8794	9.8177	5.6642	5.105	4.7042
**Security**	Mean	25.156	64.7868	93.19281	92.6511	96.927
	Standard Deviation	9.9211	9.76	6.2042	6.0099	2.8871
**Reliability**	Mean	62.4887	71.9445	73.9896	56.0163	72.1656
	Standard Deviation	10.8032	6.9778	5.294	4.3846	3.4046
**Cost**	Mean	81.7683	84.6921	81.9717	91.47	99.959
	Standard Deviation	8.6451	8.2057	7.554	8.0368	0.6703
**Ranking**	Mean	1.3519	3.6184	4.9949	4.5839	5.5569
	Standard Deviation	0.6497	0.5367	0.2777	0.4943	0.5017

**Table 8:** Clusters’ Density Characterization.

Clustered Instances	Percentage
**0**	48 (6%)
**1**	200 (23%)
**2**	269 (32%)
**3**	124 (15%)
**4**	212 (25%)
Log likelihood: −14.30591	

**Table 9:** Comparative Analysis.

Research	Accuracy
[5]	90.71%
[57]	73.02%
[58]	93.40%
Proposed (SMOreg)	98.71%.
Multilayer Perceptron	98.02%
Linear Regression	71.4%.

## Data Availability

The datasets used for this study are publicly available.
